# Supplementary material for: Health-Related Quality of Life in Oral Cancer Patients: Scoping Review and Critical Appraisal of Investigated Determinants
Source: Cancers (Basel). 2021 Aug 31;13(17):4398. doi: 10.3390/cancers13174398 (PMC8431462; doi:10.3390/cancers13174398)
Supplement: Supplementary file 1 [file cancers-13-04398-s001.zip › Table S2 (Reason for the exclusion after full-text reading).pdf]

Table S2. Reason for the exclusion after full-text reading.Legend to Table S2: OC = Oral cancer; OPC = Oropharyngeal cancer; HN = Head and Neck.

	**Study**	**Title**	**Reason for exclusion**
[1]	Abendstein 2005	Quality of life and head and neck cancer: a 5 year prospective study	Does not stratify by different HN cancers
[2]	Ackerstaff 2009	First-year quality of life assessment of an intra-arterial (RADPLAT) versus intravenous chemoradiation phase III trial	Does not stratify by different HN cancers
[3]	Ackerstaff 2012	Five-year quality of life results of the randomized clinical phase III (RADPLAT) trial, comparing concomitant intra-arterial versus intravenous chemoradiotherapy in locally advanced head and neck cancer	Does not stratify by different HN cancers
[4]	Allal 2003	Quality of life in patients with oropharynx carcinomas: Assessment after accelerated radiotherapy with or without chemotherapy versus radical surgery and postoperative radiotherapy	OPC only
[5]	Allison 1998	Correlates of health-related quality of life in upper aerodigestive tract cancer patients	Inadequate use of questionnaires
[6]	Allison 2002	Alcohol consumption is associated with improved health-related quality of life in head and neck cancer patients	Inadequate stratification by cancer site
[7]	Al Mamgani 2013	A prospective evaluation of patient-reported quality-of-life after (chemo)radiation for oropharyngeal cancer: which patients are at risk of significant quality-of-life deterioration?	OPC only
[8]	Almstahl 2016	Explorative study on quality of life in relation to salivary secretion rate in patients with head and neck cancer treated with radiotherapy	Does not stratify by different HN cancers
[9]	Almstahl 2019	Explorative study on quality of life in relation to salivary secretion rate in head and neck cancer patients treated with radiotherapy up to 2 years post treatment	Does not stratify by different HN cancers
[10]	Al-Nawas 2006	Quantifying radioxerostomia: Salivary flow rate, examiner's score, and quality of life questionnaire	Last HRQOL assessment performed before 12-month post-treatment
[11]	Antunes 2013	Phase III trial of low-level laser therapy to prevent oral mucositis in head and neck cancer patients treated with concurrent chemoradiation	Does not include OC

	Study	Title	Reason for exclusion
[12]	Artopoulou 2017	Effects of sociodemographic, treatment variables, and medical characteristics on quality of life of patients with maxillectomy restored with obturator prostheses	Inadequate cancer site description
[13]	Axelsson 2017	Effect of prophylactic percutaneous endoscopic gastrostomy tube on swallowing in advanced head and neck cancer: A randomized controlled study	Does not stratify by different HN cancers
[14]	Bachok 2018	Preliminary Comparative Study of Oral7® Versus Salt-Soda Mouthwash on Oral Health Related Problems and Quality of Life among Head and Neck Cancer Patients Undergoing Radiotherapy	Inadequate cancer site description
[15]	Baharvand 2013	Taste alteration and impact on quality of life after head and neck radiotherapy	Last HRQOL assessment performed before 12-month post-treatment
[16]	Bahig 2020	Phase II study of de-intensified intensity-modulated radiotherapy and concurrent carboplatin/5-fluorouracil in lateralized p16-associated oropharyngeal carcinoma	OPC only
[17]	Bajwa 2016	High-dose-rate interstitial brachytherapy in oral cancer-Its impact on quality of life	Does not stratify by different HN cancers
[18]	Baxi 2018	Long-term quality of life in older patients with HPV-related oropharyngeal cancer	OPC only
[19]	Beetz 2012	NTCP models for patient-rated xerostomia and sticky saliva after treatment with intensity modulated radiotherapy for head and neck cancer: the role of dosimetric and clinical factors	Does not stratify by different HN cancers
[20]	Bjordal 1994	Quality of life in patients treated for head and neck cancer: a follow-up study 7 to 11 years after radiotherapy	Does not stratify by different HN cancers
[21]	Bjordal 1995	Patient self-reported and clinician-rated quality of life in head and neck cancer patients: a cross-sectional study	Does not stratify by different HN cancers
[22]	Bjordal 2001	A prospective study of quality of life in head and neck cancer patients. Part II: Longitudinal data	Does not stratify by different HN cancers
[23]	Borggreven 2007	Quality of life and functional status in patients with cancer of the oral cavity and oropharynx: pretreatment values of a prospective study	Repeated sample

	Study	Title	Reason for exclusion
[24]	Bower 2010	Mode of treatment affects quality of life in head and neck cancer survivors: Implications for holistic care	Inadequate stratification by cancer site
[25]	Bozec 2008	Free-flap head and neck reconstruction and quality of life: a 2-year prospective study	Does not stratify by different HN cancers
[26]	Bozec 2018	Long-term quality of life and psycho-social outcomes after oropharyngeal cancer surgery and radial forearm free-flap reconstruction: A GETTEC prospective multicentric study	Does not stratify by different HN cancers
[27]	Braam 2007	Quality of life and salivary output in patients with head-and-neck cancer five years after radiotherapy	Does not stratify by different HN cancers
[28]	Broglie 2013	Quality of life of oropharyngeal cancer patients with respect to treatment strategy and p16-positivity	OPC only
[29]	Carmignani 2018	Analysis of dysphagia in advanced-stage head-and-neck cancer patients: impact on quality of life and development of a preventive swallowing treatment	Inadequate stratification by cancer site
[30]	Carrillo 2016	The impact of treatment on quality of life of patients with head and neck cancer and its association with prognosis	Does not stratify by different HN cancers
[31]	Chandu 2005	The assessment of quality of life in patients who have undergone surgery for oral cancer: a preliminary report	Last HRQOL assessment performed before 12-month post-treatment
[32]	Chen 2013	Scintigraphic assessment of salivary function after intensity-modulated radiotherapy for head and neck cancer: Correlations with parotid dose and quality of life	Does not stratify by different HN cancers
[33]	Chera 2018	Mature results of a prospective study of deintensified chemoradiotherapy for low-risk human papillomavirus-associated oropharyngeal squamous cell carcinoma	OPC only
[34]	Ch’ng 2014	Prospective quality of life assessment between treatment groups for oral cavity squamous cell carcinoma	Does not stratify by different HN cancers
[35]	Citak 2019	Nutritional status in patients with head and neck cancer undergoing radiotherapy: a longitudinal study	Does not stratify by different HN cancers

	Study	Title	Reason for exclusion
[36]	Ciuman 2007	The forearm flap: assessment of functional and aesthetic outcomes and quality of life	Last HRQOL assessment performed before 12-month post-treatment
[37]	Cnossen 2012	Computerized monitoring of patient-reported speech and swallowing problems in head and neck cancer patients in clinical practice	Does not stratify by different HN cancers
[38]	Côté 2016	Improving quality of life with nabilone during radiotherapy treatments for head and neck cancers: A randomized double-blind placebo-controlled trial	Does not stratify by different HN cancers
[39]	Curran 2007	Quality of life in head and neck cancer patients after treatment with high-dose radiotherapy alone or in combination with cetuximab	Does not include OC
[40]	Davudov 2020 a	Quality of life in patients with oral cancer treated by different reconstruction methods as measured by the EORTC QLQ-H&N43	Last HRQOL assessment performed before 12-month post-treatment
[41]	Davudov 2020 b	Psychometric evaluation of Azeri version of the head and neck cancer specific quality of life questionnaire (EORTC QLQ-H&N43)	Does not meet the rationale
[42]	De Graeff 1999	A prospective study on quality of life of patients with cancer of the oral cavity or oropharynx treated with surgery with or without radiotherapy	Does not stratify by different HN cancers
[43]	De Graeff 2000 a	Pretreatment factors predicting quality of life after treatment for head and neck cancer	Does not stratify by different HN cancers
[44]	De Graeff 2000 b	Long-term quality of life of patients with head and neck cancer	Does not stratify by different HN cancers
[45]	Derks 2003	Elderly patients with head and neck cancer: short-term effects of surgical treatment on quality of life	Last HRQOL assessment performed before 12-month post-treatment
[46]	Derks 2004	Quality of life in elderly patients with head and neck cancer one year after diagnosis	Does not stratify by different HN cancers
[47]	Derks 2005 a	Differences in coping style and locus of control between older and younger patients with head and neck cancer	Does not stratify by different HN cancers

	Study	Title	Reason for exclusion
[48]	Derks 2005 b	Reasons for non-standard treatment in elderly patients with advanced head and neck cancer	Does not stratify by different HN cancers
[49]	de Vries 2020	Frailty is associated with decline in health-related quality of life of patients treated for head and neck cancer	Does not stratify by different HN cancers
[50]	Dholam 2020	Changes in the quality of life and acoustic speech parameters of patients in various stages of prosthetic rehabilitation with an obturator after maxillectomy	Last HRQOL assessment performed before 12-month post-treatment
[51]	Do 2018	Comparison of hospital based and home based exercise on quality of life, and neck and shoulder function in patients with spinal accessary nerve injury after head and neck cancer surgery	Does not stratify by different HN cancers
[52]	Driessen 2018	Quality of life of patients with locally advanced head and neck cancer treated with induction chemotherapy followed by cisplatin-containing chemoradiotherapy in the Dutch CONDOR study: a randomized controlled trial	Does not stratify by different HN cancers
[53]	Duncan 2005	Quality of life, mucositis, and xerostomia from radiotherapy for head and neck cancers: A report from the NCIC CTG HN2 randomized trial of an antimicrobial lozenge to prevent mucositis	Does not stratify by different HN cancers
[54]	Dziegielewski 2013	Total glossectomy with laryngeal preservation and free flap reconstruction: Objective functional outcomes and systematic review of the literature	Inadequate use of questionnaires
[55]	Dziegielewski 2019	Beavertail modification of the radial forearm free flap in total oral glossectomy reconstruction: Technique and functional outcomes	Inadequate use of questionnaires
[56]	Egestad 2015	Differences in quality of life in obese and normal weight head and neck cancer patients undergoing radiation therapy	Does not stratify by different HN cancers
[57]	Elaldi 2020	Correlations between long-term quality of life and patient needs and concerns following head and neck cancer treatment and the impact of psychological distress. A multicentric cross-sectional study	Does not stratify by different HN cancers

	Study	Title	Reason for exclusion
[58]	Elfring 2014	The relationship between lingual and hypoglossal nerve function and quality of life in head and neck cancer	OPC only
[59]	Epstein 1999	Quality of life and oral function following radiotherapy for head and neck cancer	Inadequate use of questionnaires
[60]	Epstein 2001	Quality of life and oral function in patients treated with radiation therapy for head and neck cancer	Does not stratify by different HN cancers
[61]	Erkal 2014	Assessment of early and late dysphagia using videofluoroscopy and quality of life questionnaires in patients with head and neck cancer treated with radiation therapy	Does not include OC
[62]	Ettl 2016	Impact of radiotherapy on implant-based prosthetic rehabilitation in patients with head and neck cancer: A prospective observational study on implant survival and quality of life—Preliminary results	Does not stratify by different HN cancers
[63]	Evensen 2002	Side effects and quality of life after inadvertent radiation overdosage in brachytherapy of head-and-neck cancer	Does not stratify by different HN cancers
[64]	Fang 2004	Changes in quality of life of head-and-neck cancer patients following postoperative radiotherapy	Does not stratify by different HN cancers
[65]	Fang 2005	Changing quality of life in patients with advanced head and neck cancer after primary radiotherapy or chemoradiation	Does not stratify by different HN cancers
[66]	Felser 2020	Feasibility and Effects of a Supervised Exercise Program Suitable for Independent Training at Home on Physical Function and Quality of Life in Head and Neck Cancer Patients: A Pilot Study	Does not stratify by different HN cancers
[67]	Fierz 2013	Patients with oral tumors. Part 2: Quality of life after treatment with resection prostheses. Resection prosthetics: evaluation of quality of life	Inadequate cancer site description
[68]	Franco 2017	Management of acute skin toxicity with Hypericum perforatum and neem oil during platinum-based concurrent chemo-radiation in head and neck cancer patients	Does not stratify by different HN cancers
[69]	Funk 2014	A randomized clinical trial to evaluate the impact of a dental care program in the quality of life of head and neck cancer patients	Inadequate cancer site description

	Study	Title	Reason for exclusion
[70]	Galitis 2017	Trismus and reduced quality of life in patients with oral squamous cell carcinoma, who received post-operative radiotherapy alone or combined with chemotherapy	Last HRQOL assessment performed before 12-month post-treatment
[71]	Gandhi 2014	Symptom Burden and Quality of Life in Advanced Head and Neck Cancer Patients: AIIMS Study of 100 Patients	Inadequate cancer site description
[72]	Goetz 2020	Hospital based quality of life in oral cancer surgery	Last HRQOL assessment performed before 12-month post-treatment
[73]	Goiato 2020	The Impact of Surgery and Radiotherapy on Health-Related Quality of Life of Individuals with Oral and Oropharyngeal Carcinoma and Short-Term Follow up after Treatment	Last HRQOL assessment performed before 12-month post-treatment
[74]	Guenzel 2018	The Impact of Surgery and Radiotherapy on Health-Related Quality of Life of Individuals with Oral and Oropharyngeal Carcinoma and Short-Term Follow up after Treatment	OPC only
[75]	Ham 2019	Health-related quality of life of patients treated with chemoradiotherapy plus or minus prophylactic antibiotics to reduce the number of pneumonias for locally advanced head and neck cancer, the PANTAP study	Does not stratify by different HN cancers
[76]	Hammerlid 1997 a	Prospective, longitudinal quality-of-life study of patients with head and neck cancer: A feasibility study including the EORTC QLC-C30	Does not stratify by different HN cancers
[77]	Hammerlid 1997 b	A prospective quality of life study of patients with oral or pharyngeal carcinoma treated with external beam irradiation with or without brachytherapy	Does not stratify by different HN cancers
[78]	Hammerlid 2001 a	A prospective study of quality of life in head and neck cancer patients. Part I: At diagnosis	Does not stratify by different HN cancers
[79]	Hammerlid 2001 b	Health-related quality of life three years after diagnosis of head and neck cancer--a longitudinal study	Does not stratify by different HN cancers
[80]	Hammerlid 2001 c	Health-related quality of life in long-term head and neck cancer survivors: a comparison with general population norms	Does not stratify by different HN cancers
[81]	Hashemipour 2020	Quality of life in Iranian patients with head-and-neck cancer	Last HRQOL assessment performed before 12-month post-treatment

	Study	Title	Reason for exclusion
[82]	Hassanein 2005	Psychological outcome of patients following treatment of oral cancer and its relation with functional status and coping mechanisms	Last HRQOL assessment performed before 12-month post-treatment
[83]	Hermann 2020	A single-institution, randomized, pilot study evaluating the efficacy of gabapentin and methadone for patients undergoing chemoradiation for head and neck squamous cell cancer	Does not stratify by different HN cancers
[84]	Ho 2010	Comparison of patient-reported late treatment toxicity (LENT-SOMA) with quality of life (EORTC QLQ-C30 and QLQ-H&N35) assessment after head and neck radiotherapy	Does not stratify by different HN cancers
[85]	Horn 2020	Prospective feasibility analysis of salvage surgery in recurrent oral cancer in terms of quality of life	Last HRQOL assessment performed before 12-month post-treatment
[86]	Huguenin 1999	Quality of life in patients cured from a carcinoma of the head and neck by radiotherapy: the importance of the target volume	Does not stratify by different HN cancers
[87]	Infante-Cossio 2009	Quality of life in patients with oral and oropharyngeal cancer	Does not meet the rationale
[88]	Iwase 2003	Hypercalcemic complication in patients with oral squamous cell carcinoma	Last HRQOL assessment performed before 12-month post-treatment
[89]	Jacobsen 2016	Oral rehabilitation with dental implants and quality of life following mandibular reconstruction with free fibular flap	Includes non-cancer patients
[90]	Jellema 2005	Does radiation dose to the salivary glands and oral cavity predict patient-rated xerostomia and sticky saliva in head and neck cancer patients treated with curative radiotherapy?	Does not stratify by different HN cancers
[91]	Jellema 2007	Unilateral versus bilateral irradiation in squamous cell head and neck cancer in relation to patient-rated xerostomia and sticky saliva	Does not stratify by different HN cancers
[92]	Jenewein 2008	Quality of life and dyadic adjustment in oral cancer patients and their female partners	Last HRQOL assessment performed before 12-month post-treatment
[93]	Jensen 2006 a	The relationship between observer-based toxicity scoring and patient assessed symptom severity after treatment for head and neck cancer. A correlative cross sectional study of the DAHANCA toxicity scoring system and the EORTC quality of life questionnaires	Last HRQOL assessment performed before 12-month post-treatment

	Study	Title	Reason for exclusion
[94]	Jensen 2006 b	A cross sectional quality of life study of 116 recurrence free head and neck cancer patients. The first use of EORTC H&N35 in Danish	Last HRQOL assessment performed before 12-month post-treatment
[95]	Jensen 2007	Smoking has a negative impact upon health related quality of life after treatment for head and neck cancer	Last HRQOL assessment performed before 12-month post-treatment
[96]	Kaae 2019	Relationship between patient and physician-rated xerostomia and dose distribution to the oral cavity and salivary glands for head and neck cancer patients after radiotherapy	Insufficient OC sample
[97]	Kermer 2004	Two stage reconstruction with revascularized grafts after resection of retromolar and oropharyngeal carcinoma	Does not stratify by different HN cancers
[98]	Kim 2016	Pretreatment depression as a prognostic indicator of survival and nutritional status in patients with head and neck cancer	Does not stratify by different HN cancers
[99]	Kjeldsted 2020	Association between human papillomavirus status and health-related quality of life in oropharyngeal and oral cavity cancer survivors	Last HRQOL assessment performed before 12-month post-treatment
[100]	Korfage 2010	Five-year follow-up of oral functioning and quality of life in patients with oral cancer with implant-retained mandibular overdentures	Does not stratify by different HN cancers
[101]	Korsten 2021	The course of health-related quality of life from diagnosis to two years follow-up in patients with oropharyngeal cancer: does HPV status matter?	OPC only
[102]	Krebber 2016	Screening for psychological distress in follow-up care to identify head and neck cancer patients with untreated distress	Last HRQOL assessment performed before 12-month post-treatment
[103]	Kumar 2016	Implant supported dental rehabilitation following segmental mandibular reconstruction-quality of life outcomes of a prospective randomized trial	Includes non-cancer patients
[104]	Lal 2018	Objective and subjective assessment of xerostomia in patients of locally advanced head-and-neck cancers treated by intensity-modulated radiotherapy	Does not stratify by different HN cancers
[105]	Landstrom 2015	Long-term follow-up in patients treated with curative electrochemotherapy for cancer in the oral cavity and oropharynx	Does not stratify by different HN cancers

	Study	Title	Reason for exclusion
[106]	Lazaridou 2016	Nasolabial pedicled compared with island flaps for intraoral reconstruction of oncological defects: complications, recovery of sensitivity, and assessment of quality of life	Last HRQOL assessment performed before 12-month post-treatment
[107]	Lazarus 2013	Tongue strength as a predictor of functional outcomes and quality of life after tongue cancer surgery	Last HRQOL assessment performed before 12-month post-treatment
[108]	Lazarus 2014	Functional outcomes and quality of life after chemoradiotherapy: Baseline and 3 and 6 months post-treatment	Last HRQOL assessment performed before 12-month post-treatment
[109]	Lee 2010	Prospective changes of the quality of life for patients newly diagnosed with oral cancer during the acute stage	Last HRQOL assessment performed before 12-month post-treatment
[110]	Lee 2015	Postradiation trismus and its impact on quality of life in patients with head and neck cancer	Does not stratify by different HN cancers
[111]	Likhterov 2018	Objective and subjective hyposalivation after treatment for head and neck cancer: Long-term outcomes	Does not stratify by different HN cancers
[112]	Lima 2010	Efficacy of low-level laser therapy and aluminum hydroxide in patients with chemotherapy and radiotherapy-induced oral mucositis	Does not stratify by different HN cancers
[113]	Linkblom 2014	Radiation-induced trismus in the ARTSCAN head and neck trial	Does not stratify by different HN cancers
[114]	Llewellyn 2006	Head and neck cancer: To what extent can psychological factors explain differences between health-related quality of life and individual quality of life?	Does not meet the rationale
[115]	Llewellyn 2007	Illness and treatment beliefs in head and neck cancer: Is Leventhal's common sense model a useful framework for determining changes in outcomes over time?	Last HRQOL assessment performed before 12-month post-treatment
[116]	Lofstrand 2018	Quality of Life after Free Fibula Flap Reconstruction of Segmental Mandibular Defects	Last HRQOL assessment performed before 12-month post-treatment
[117]	Lopez-Jornet 2012	Assessing quality of life in patients with head and neck cancer in Spain by means of EORTC QLQ-C30 and QLQ-H&N35	Last HRQOL assessment performed before 12-month post-treatment
[118]	Louise-Kent 2008	Radiation-induced trismus in head and neck cancer patients	Inadequate use of questionnaires

	Study	Title	Reason for exclusion
[119]	Low 2009	Issues of intimacy and sexual dysfunction following major head and neck cancer treatment	Inadequate use of questionnaires
[120]	Maciewski 2010	Gender specific quality of life in patients with oral squamous cell carcinomas	Last HRQOL assessment performed before 12-month post-treatment
[121]	Melissant 2018	The course of sexual interest and enjoyment in head and neck cancer patients treated with primary (chemo)radiotherapy	Inadequate use of questionnaires
[122]	Milovanovic 2019	The impact of socioeconomic factors on quality of life and functional impairment in patients treated for oropharyngeal carcinoma	OPC only
[123]	Mogadas 2020	Influence of radiation dose to pharyngeal constrictor muscles on late dysphagia and quality of life in patients with locally advanced oropharyngeal carcinoma	OPC only
[124]	Nagy 2014	Quality of life in head and neck cancer patients after tumor therapy and subsequent rehabilitation: An exploratory study	Does not stratify by different HN cancers
[125]	Ninu 2016	Psychological distress and health-related quality of life among head and neck cancer patients during the first year after treatment	Last HRQOL assessment performed before 12-month post-treatment
[126]	Noronha 2016	Health-related quality of life in patients with metastatic, relapsed, or inoperable squamous cell carcinoma of the head and neck in India	Does not stratify by different HN cancers
[127]	Nourissat 2012	Factors associated with weight loss during radiotherapy in patients with stage i or II head and neck cancer	Does not stratify by different HN cancers
[128]	Nyqvist 2016	Differences in health related quality of life in the randomised ARTSCAN study; Accelerated vs. conventional radiotherapy for head and neck cancer. A five year follow up	Does not stratify by different HN cancers
[129]	Oates 2014	The effect of cancer stage and treatment modality on quality of life in oropharyngeal cancer	OPC only
[130]	Ohrn 2001	Oral health and quality of life among patients with head and neck cancer or haematological malignancies	Includes non-cancer patients
[131]	Oliveira 2014	Influence of pain severity on the quality of life in patients with head and neck cancer before antineoplastic therapy	Does not stratify by different HN cancers

	Study	Title	Reason for exclusion
[132]	Oskam 2010	Quality of life as predictor of survival: A prospective study on patients treated with combined surgery and radiotherapy for advanced oral and oropharyngeal cancer	Last HRQOL assessment performed before 12-month post-treatment
[133]	Osthus 2011	Head and neck specific Health Related Quality of Life scores predict subsequent survival in successfully treated head and neck cancer patients: A prospective cohort study	Does not stratify by different HN cancers
[134]	Passchier 2016	Feasibility and impact of a dedicated multidisciplinary rehabilitation program on health-related quality of life in advanced head and neck cancer patients	Does not stratify by different HN cancers
[135]	Pauli 2013	The incidence of trismus and long-term impact on health-related quality of life in patients with head and neck cancer	Does not stratify by different HN cancers
[136]	Pauli 2014	Exercise intervention for the treatment of trismus in head and neck cancer	Insufficient OC sample
[137]	Pauli 2015	Treating trismus: A prospective study on effect and compliance to jaw exercise therapy in head and neck cancer	Insufficient OC sample
[138]	Pauli 2016	Exercise intervention for the treatment of trismus in head and neck cancer - a prospective two-year follow-up study	Insufficient OC sample
[139]	Petrovic 2019	Long-term functional and esthetic outcomes after fibula free flap reconstruction of the mandible	Includes non-cancer patients
[140]	Pierre 2014	Long-term functional outcomes and quality of life after oncologic surgery and microvascular reconstruction in patients with oral or oropharyngeal cancer	Repeated sample
[141]	Pollom 2015	A prospective study of electronic quality of life assessment using tablet devices during and after treatment of head and neck cancers	Does not stratify by different HN cancers
[142]	Ranta 2020	Long-term Quality of Life After Treatment of Oropharyngeal Squamous Cell Carcinoma	OPC only
[143]	Rathod 2013	Quality-of-life (QOL) outcomes in patients with head and neck squamous cell carcinoma (HNSCC) treated with intensity-modulated radiation therapy (IMRT) compared to three-dimensional conformal radiotherapy (3D-CRT): Evidence from a prospective randomized study	Does not include OC

	Study	Title	Reason for exclusion
[144]	Ravasco 2005	Impact of nutrition on outcome: a prospective randomized controlled trial in patients with head and neck cancer undergoing radiotherapy	Does not stratify by different HN cancers
[145]	Rhemrev 2007	Long-term functional outcome and satisfaction after radial forearm free flap reconstructions of intraoral malignancy resections	Does not stratify by different HN cancers
[146]	Rinkel 2009	The psychometric and clinical validity of the SWAL-QOL questionnaire in evaluating swallowing problems experienced by patients with oral and oropharyngeal cancer	Does not meet the rationale
[147]	Rogers 1998	A comparison between the University of Washington Head and Neck Disease- Specific Measure and the Medical Short Form 36, EORTC QOQ-C33 and EORTC Head and Neck 35	Does not stratify by different HN cancers
[148]	Rogers 1999	Quality of life 5-10 years after primary surgery for oral and oro-pharyngeal cancer	Does not stratify by different HN cancers
[149]	Rogers 2000	Distinct patient groups in oral cancer: A prospective study of perceived health status following primary surgery	Does not stratify by different HN cancers
[150]	Rogers 2001	The relationship between length of stay and health-related quality of life in patients treated by primary surgery for oral and oropharyngeal cancer	Does not stratify by different HN cancers
[151]	Rogers 2020	Quality of life, cognitive, physical and emotional function at diagnosis predicts head and neck cancer survival: analysis of cases from the Head and Neck 5000 study	Does not meet the rationale
[152]	Roick 2019	Predictors of changes in quality of life in head and neck cancer patients: a prospective study over a 6-month period	Last HRQOL assessment performed before 12-month post-treatment
[153]	Ruhl 1997	Survival, function, and quality of life after total glossectomy	Does not stratify by different HN cancers
[154]	Ryzek 2014	Early stage oropharyngeal carcinomas: Comparing quality of life for different treatment modalities	OPC only
[155]	Sandmæl 2020	Physical rehabilitation in patients with head and neck cancer: Impact on health-related quality of life and suitability of a post-treatment program	Insufficient OC sample

	Study	Title	Reason for exclusion
[156]	Schiefke 2009	Function, postoperative morbidity, and quality of life after cervical sentinel node biopsy and after selective neck dissection	Does not stratify by different HN cancers
[157]	Schliephake 1998	Speech, deglutition and life quality after intraoral tumour resection: A prospective study	Does not stratify by different HN cancers
[158]	Schliephake 2002 a	Prospective evaluation of quality of life after oncologic surgery for oral cancer	Does not use EORTC questionnaires
[159]	Schliephake 2002 b	Impact of intraoral soft-tissue reconstruction on the development of quality of life after ablative surgery in patients with oral cancer	Does not stratify by different HN cancers
[160]	Scott 2020	Long-term quality of life & functional outcomes after treatment of oropharyngeal cancer	OPC only
[161]	Shepherd 2004	Prospective evaluation of quality of life in patients with oral and oropharyngeal cancer: From diagnosis to three months post-treatment	Does not stratify by different HN cancers
[162]	Silveira 2011	Geriatric oncology: Comparing health related quality of life in head and neck cancer patients	Does not stratify by different HN cancers
[163]	Sjamsudin 2018	Assessment of oral cancer pain, anxiety, and quality of life of oral squamous cell carcinoma patients with invasive treatment procedure	Last HRQOL assessment performed before 12-month post-treatment
[164]	Sodergren 2018	Does age matter? A comparison of health-related quality of life issues of adolescents and young adults with cancer	Does not stratify by different HN cancers
[165]	Soderstrom 2016	Dysphagia – Results from multivariable predictive modelling on aspiration from a subset of the ARTSCAN trial	Does not meet the rationale
[166]	Spiegel 2019	Quality of life in patients after reconstruction with the supraclavicular artery island flap (SCAIF) versus the radial free forearm flap (RFFF)	Does not stratify by different HN cancers
[167]	Stromgren 2004	Pain characteristics and treatment outcome for advanced cancer patients during the first week of specialized palliative care	Does not stratify by different HN cancers
[168]	Subramanian 2018	Health-related quality of life and psychological distress among cancer survivors in a middle-income country	Does not stratify by different HN cancers

	Study	Title	Reason for exclusion
[169]	Suzuki 2016	Anxiety and depression in patients after surgery for head and neck cancer in Japan	Does not stratify by different HN cancers
[170]	Teguh 2009	Early Hyperbaric Oxygen Therapy for Reducing Radiotherapy Side Effects: Early Results of a Randomized Trial in Oropharyngeal and Nasopharyngeal Cancer	Does not include OC
[171]	Tesic 2020	Validation of the oral health impact profile - 14 in patients with head and neck cancer	Does not meet the rationale
[172]	Thor 2017	Temporal patterns of patient-reported trismus and associated mouth-opening distances in radiotherapy for head and neck cancer: A prospective cohort study	Includes non-cancer patients
[173]	Tribius 2012	Global quality of life during the acute toxicity phase of multimodality treatment for patients with head and neck cancer: Can we identify patients most at risk of profound quality of life decline?	Does not stratify by different HN cancers
[174]	Tribius 2015	Residual deficits in quality of life one year after intensity-modulated radiotherapy for patients with locally advanced head and neck cancer: Results of a prospective study	Last HRQOL assessment performed before 12-month post-treatment
[175]	Tribius 2018	Socioeconomic status and quality of life in patients with locally advanced head and neck cancer	Does not stratify by different HN cancers
[176]	Tribius 2020	Prognostic factors for lymphedema in patients with locally advanced head and neck cancer after combined radio(chemo)therapy- results of a longitudinal study	Does not use EORTC questionnaires
[177]	Tribius 2021	Parotid sparing and quality of life in long-term survivors of locally advanced head and neck cancer after intensity-modulated radiation therapy	Does not stratify by different HN cancers
[178]	Tschiersner 2010	Content validation of the International Classification of Functioning, Disability and Health core sets for head and neck cancer: A multicentre study	Does not stratify by different HN cancers
[179]	Tschiersner 2012	Functional outcome in patients with advanced head and neck cancer: Surgery and reconstruction with free flaps versus primary radiochemotherapy	Inadequate use of questionnaires
[180]	Van Beek 2020	The course of symptoms of anxiety and depression from time of diagnosis up to 2 years follow-up in head and neck cancer patients treated with primary (chemo)radiation	Does not stratify by different HN cancers

	Study	Title	Reason for exclusion
[181]	Van Cann 2005	Health related quality of life after mandibular resection for oral and oropharyngeal squamous cell carcinoma	Does not stratify by different HN cancers
[182]	van den berg 2008	A prospective study on malnutrition and quality of life in patients with head and neck cancer	Does not stratify by different HN cancers
[183]	van der Meulen 2014	Long-term effect of a nurse-led psychosocial intervention on health-related quality of life in patients with head and neck cancer: a randomised controlled trial	Does not stratify by different HN cancers
[184]	van der Schroeff 2007	The effect of age on survival and quality of life in elderly head and neck cancer patients: A long-term prospective study	Does not stratify by different HN cancers
[185]	van Nieuwenhuizen 2017	Patient-reported physical activity and the association with health-related quality of life in head and neck cancer survivors	Does not stratify by different HN cancers
[186]	Veldhuis 2016	Tumor site and disease stage as predictors of quality of life in head and neck cancer: a prospective study on patients treated with surgery or combined therapy with surgery and radiotherapy or radiochemotherapy	Does not include OC
[187]	Verdonk-de Leeuw 2009	Computerized prospective screening for high levels of emotional distress in head and neck cancer patients and referral rate to psychosocial care	Does not stratify by different HN cancers
[188]	Verdonk-de Leeuw 2010	Employment and return to work in head and neck cancer survivors	Does not stratify by different HN cancers
[189]	Verdonk-de Leeuw 2014	The course of health-related quality of life in head and neck cancer patients treated with chemoradiation: A prospective cohort study	Does not stratify by different HN cancers
[190]	Villaron 2018	Telehealth applied to physical activity during cancer treatment: a feasibility, acceptability, and randomized pilot study	Insufficient OC sample
[191]	Wang 2016	Impact of post-chemoradiotherapy superselective/selective neck dissection on patient reported quality of life	OPC only

	Study	Title	Reason for exclusion
[192]	Wang 2017	Functional outcome and quality of life after a maxillectomy: a comparison between an implant supported obturator and implant supported fixed prostheses in a free vascularized flap	Does not stratify by different HN cancers
[193]	Wan Leung 2011	Health-related Quality of life in 640 head and neck cancer survivors after radiotherapy using EORTC QLQ-C30 and QLQ-H&N35 questionnaires	Repeated sample
[194]	Watkinson 2002	Conservation surgery in the management of T1 and T2 oropharyngeal squamous cell carcinoma: The Birmingham UK experience	OPC only
[195]	Yan 2018	The association of tooth loss, toothbrushing, and quality of life among cancer survivors	Does not stratify by different HN cancers
[196]	Yokota 2018	A phase II study of HMB/Arg/Gln against oral mucositis induced by chemoradiotherapy for patients with head and neck cancer	Does not stratify by different HN cancers
[197]	You 2020	Comparison of functional outcomes and health-related quality of life one year after treatment in patients with oral and oropharyngeal cancer treated with three different reconstruction methods	Does not stratify by different HN cancers
[198]	Znrnc 2020	Complex mandibular reconstruction for head and neck squamous cell carcinoma—the ongoing challenge in reconstruction and rehabilitation	Last HRQOL assessment performed before 12-month post-treatment

REFERENCES

1. Abendstein H, Nordgren, M, Boysen, M, et al. Quality of life and head and neck cancer: a 5 year prospective study. Laryngoscope 2005;115:2183-92.
2. Ackerstaff AH, Balm, AJ, Rasch, CR, et al. First-year quality of life assessment of an intra-arterial (RADPLAT) versus intravenous chemoradiation phase III trial. Head Neck 2009;31:77-84.
3. Ackerstaff AH, Rasch, CR, Balm, AJ, et al. Five-year quality of life results of the randomized clinical phase III (RADPLAT) trial, comparing concomitant intra-arterial versus intravenous chemoradiotherapy in locally advanced head and neck cancer. Head Neck 2012;34:974-80.
4. Allal AS, Nicoucar, K, Mach, N, et al. Quality of life in patients with oropharynx carcinomas: Assessment after accelerated radiotherapy with or without chemotherapy versus radical surgery and postoperative radiotherapy. Head and Neck 2003;25:833-40.

5. Allison PJ, Locker, D, Wood-Dauphinee, S, et al. Correlates of health-related quality of life in upper aerodigestive tract cancer patients. Quality of Life Research 1998;7:713-22.
6. Allison PJ. Alcohol consumption is associated with improved health-related quality of life in head and neck cancer patients. Oral Oncol 2002;38:81-6.
7. Al-Mamgani A, van Rooij, P, Tans, L, et al. A prospective evaluation of patient-reported quality-of-life after (chemo)radiation for oropharyngeal cancer: which patients are at risk of significant quality-of-life deterioration? Radiother Oncol 2013;106:359-63.
8. Almstähl A, Alstad, T, Fagerberg-Mohlin, B, et al. Explorative study on quality of life in relation to salivary secretion rate in patients with head and neck cancer treated with radiotherapy. Head and Neck 2016;38:782-91.
9. Almståhl A, Skoogh Andersson, J, Alstad, T, et al. Explorative study on quality of life in relation to salivary secretion rate in head and neck cancer patients treated with radiotherapy up to 2 years post treatment. International journal of dental hygiene 2019;17:46-54.
10. Al-Nawas B, Al-Nawas, K, Kunkel, M, et al. Quantifying radioxerostomia: Salivary flow rate, examiner's score, and quality of life questionnaire. Strahlentherapie und Onkologie 2006;182:336-41.
11. Antunes HS, Herchenhorn, D, Small, IA, et al. Phase III trial of low-level laser therapy to prevent oral mucositis in head and neck cancer patients treated with concurrent chemoradiation. Radiother Oncol 2013;109:297-302.
12. Artopoulou II, Karademas, EC, Papadogeorgakis, N, et al. Effects of sociodemographic, treatment variables, and medical characteristics on quality of life of patients with maxillectomy restored with obturator prostheses. The Journal of prosthetic dentistry 2017;118:783-9.
13. Axelsson L, Silander, E, Nyman, J, et al. Effect of prophylactic percutaneous endoscopic gastrostomy tube on swallowing in advanced head and neck cancer: A randomized controlled study. Head and Neck 2017;39:908-15.
14. Bachok N, Biswal, BM, Razak, NHA, et al. Preliminary Comparative Study of Oral7® Versus Salt-Soda Mouthwash on Oral Health Related Problems and Quality of Life among Head and Neck Cancer Patients Undergoing Radiotherapy. Malays J Med Sci 2018;25:79-87.
15. Baharvand M, Shoalehsaadi, N, Barakian, R, et al. Taste alteration and impact on quality of life after head and neck radiotherapy. Journal of Oral Pathology and Medicine 2013;42:106-12.
16. Bahig H, Lambert, L, Filion, E, et al. Phase II study of de-intensified intensity-modulated radiotherapy and concurrent carboplatin/5-fluorouracil in lateralized p16-associated oropharyngeal carcinoma. Head and Neck 2020;42:3479-89.
17. Bajwa HK, Singareddy, R, Alluri, KR. High-dose-rate interstitial brachytherapy in oral cancer-Its impact on quality of life. Brachytherapy 2016;15:381-6.

18. Baxi SS, Cullen, G, Xiao, H, et al. Long-term quality of life in older patients with HPV-related oropharyngeal cancer. Head and Neck 2018;40:2321-8.
19. Beetz I, Schilstra, C, van der Schaaf, A, et al. NTCP models for patient-rated xerostomia and sticky saliva after treatment with intensity modulated radiotherapy for head and neck cancer: the role of dosimetric and clinical factors. Radiother Oncol 2012;105:101-6.
20. Bjordal K, Kaasa, S, Mastekaasa, A. Quality of life in patients treated for head and neck cancer: a follow-up study 7 to 11 years after radiotherapy. Int J Radiat Oncol Biol Phys 1994;28:847-56.
21. Bjordal K, Freng, A, Thorvik, J, et al. Patient self-reported and clinician-rated quality of life in head and neck cancer patients: a cross-sectional study. Eur J Cancer B Oral Oncol 1995;31b:235-41.
22. Bjordal K, Ahlner-Elmqvist, M, Hammerlid, E, et al. A prospective study of quality of life in head and neck cancer patients. Part II: Longitudinal data. Laryngoscope 2001;111:1440-52.
23. Borggreven PA, Verdonck-de Leeuw, IM, Muller, MJ, et al. Quality of life and functional status in patients with cancer of the oral cavity and oropharynx: pretreatment values of a prospective study. Eur Arch Otorhinolaryngol 2007;264:651-7.
24. Bower WF, Vlantis, AC, Chung, TM, et al. Mode of treatment affects quality of life in head and neck cancer survivors: Implications for holistic care. Acta Otolaryngol 2010;130:1185-92.
25. Bozec A, Poissonnet, G, Chamorey, E, et al. Free-flap head and neck reconstruction and quality of life: a 2-year prospective study. Laryngoscope 2008;118:874-80.
26. Bozec A, Demez, P, Gal, J, et al. Long-term quality of life and psycho-social outcomes after oropharyngeal cancer surgery and radial forearm free-flap reconstruction: A GETTEC prospective multicentric study. Surgical Oncology 2018;27:23-30.
27. Braam PM, Roesink, JM, Raaijmakers, CP, et al. Quality of life and salivary output in patients with head-and-neck cancer five years after radiotherapy. Radiat Oncol 2007;2:3.
28. Broglie MA, Soltermann, A, Haile, SR, et al. Quality of life of oropharyngeal cancer patients with respect to treatment strategy and p16-positivity. Laryngoscope 2013;123:164-70.
29. Carmignani I, Locatello, LG, Desideri, I, et al. Analysis of dysphagia in advanced-stage head-and-neck cancer patients: impact on quality of life and development of a preventive swallowing treatment. European Archives of Oto-Rhino-Laryngology 2018;275:2159-67.
30. Carrillo JF, Carrillo, LC, Ramirez-Ortega, MC, et al. The impact of treatment on quality of life of patients with head and neck cancer and its association with prognosis. Eur J Surg Oncol 2016;42:1614-21.

31. Chandu A, Sun, KC, DeSilva, RN, et al. The assessment of quality of life in patients who have undergone surgery for oral cancer: a preliminary report. J Oral Maxillofac Surg 2005;63:1606-12.
32. Chen WC, Lai, CH, Lee, TF, et al. Scintigraphic assessment of salivary function after intensity-modulated radiotherapy for head and neck cancer: Correlations with parotid dose and quality of life. Oral Oncology 2013;49:42-8.
33. Chera BS, Amdur, RJ, Tepper, JE, et al. Mature results of a prospective study of deintensified chemoradiotherapy for low-risk human papillomavirus-associated oropharyngeal squamous cell carcinoma. Cancer 2018;124:2347-54.
34. Ch'ng S, Oates, J, Gao, K, et al. Prospective quality of life assessment between treatment groups for oral cavity squamous cell carcinoma. Head and Neck 2014;36:834-40.
35. Citak E, Tulek, Z, Uzel, O. Nutritional status in patients with head and neck cancer undergoing radiotherapy: a longitudinal study. Supportive Care in Cancer 2019;27:239-47.
36. Ciuman R, Mohr, C, Kröger, K, et al. The forearm flap: assessment of functional and aesthetic outcomes and quality of life. American Journal of Otolaryngology - Head and Neck Medicine and Surgery 2007;28:367-74.
37. Cnossen IC, de Bree, R, Rinkel, RN, et al. Computerized monitoring of patient-reported speech and swallowing problems in head and neck cancer patients in clinical practice. Support Care Cancer 2012;20:2925-31.
38. Côté M, Trudel, M, Wang, C, et al. Improving quality of life with nabilone during radiotherapy treatments for head and neck cancers: A randomized double-blind placebo-controlled trial. Annals of Otology, Rhinology and Laryngology 2016;125:317-24.
39. Curran D, Giralt, J, Harari, PM, et al. Quality of life in head and neck cancer patients after treatment with high-dose radiotherapy alone or in combination with cetuximab. Journal of Clinical Oncology 2007;25:2191-7.
40. Davudov MM, Harirchi, I, Arabkheradmand, A, et al. Quality of life in patients with oral cancer treated by different reconstruction methods as measured by the EORTC QLQ-H&N43. British Journal of Oral and Maxillofacial Surgery 2020. http://dx.doi.org/10.1016/j.bjoms.2020.06.027.
41. Davudov MM, Rahimov, C, Harirchi, I, et al. Psychometric evaluation of Azeri version of the head and neck cancer specific quality of life questionnaire (EORTC QLQ-H&N43). Health and Quality of Life Outcomes 2020;18.
42. de Graeff A, de Leeuw, JR, Ros, WJ, et al. A prospective study on quality of life of patients with cancer of the oral cavity or oropharynx treated with surgery with or without radiotherapy. Oral Oncol 1999;35:27-32.
43. de Graeff A, de Leeuw, JR, Ros, WJ, et al. Pretreatment factors predicting quality of life after treatment for head and neck cancer. Head Neck 2000;22:398-407.

44. de Graeff A, de Leeuw, JR, Ros, WJ, et al. Long-term quality of life of patients with head and neck cancer. Laryngoscope 2000;110:98-106.
45. Derks W, De Leeuw, JR, Hordijk, GJ, et al. Elderly patients with head and neck cancer: short-term effects of surgical treatment on quality of life. Clin Otolaryngol Allied Sci 2003;28:399-405.
46. Derks W, De Leeuw, RJ, Hordijk, GJ, et al. Quality of life in elderly patients with head and neck cancer one year after diagnosis. Head and Neck 2004;26:1045-52.
47. Derks W, De Leeuw, JRJ, Hordijk, GJ, et al. Differences in coping style and locus of control between older and younger patients with head and neck cancer. Clinical Otolaryngology 2005;30:186-92.
48. Derks W, De Leeuw, JRJ, Hordijk, GJ, et al. Reasons for non-standard treatment in elderly patients with advanced head and neck cancer. European Archives of Oto-Rhino-Laryngology 2005;262:21-6.
49. de Vries J, Bras, L, Sidorenkov, G, et al. Frailty is associated with decline in health-related quality of life of patients treated for head and neck cancer. Oral Oncol 2020;111:105020.
50. Dholam KP, Bachher, G, Gurav, SV. Changes in the quality of life and acoustic speech parameters of patients in various stages of prosthetic rehabilitation with an obturator after maxillectomy. J Prosthet Dent 2020;123:355-63.
51. Do JH, Yoon, IJ, Cho, YK, et al. Comparison of hospital based and home based exercise on quality of life, and neck and shoulder function in patients with spinal accessary nerve injury after head and neck cancer surgery. Oral Oncology 2018;86:100-4.
52. Driessen CML, Groenewoud, JMM, de Boer, JP, et al. Quality of life of patients with locally advanced head and neck cancer treated with induction chemotherapy followed by cisplatin-containing chemoradiotherapy in the Dutch CONDOR study: a randomized controlled trial. Supportive Care in Cancer 2018;26:1233-42.
53. Duncan GG, Epstein, JB, Tu, D, et al. Quality of life, mucositis, and xerostomia from radiotherapy for head and neck cancers: A report from the NCIC CTG HN2 randomized trial of an antimicrobial lozenge to prevent mucositis. Head and Neck 2005;27:421-8.
54. Dziegielewski PT, Ho, ML, Rieger, J, et al. Total glossectomy with laryngeal preservation and free flap reconstruction: Objective functional outcomes and systematic review of the literature. Laryngoscope 2013;123:140-5.
55. Dziegielewski PT, Rieger, J, Shama, MA, et al. Beavertail modification of the radial forearm free flap in total oral glossectomy reconstruction: Technique and functional outcomes. Oral Oncology 2019;96:71-6.
56. Egestad H, Nieder, C. Differences in quality of life in obese and normal weight head and neck cancer patients undergoing radiation therapy. Support Care Cancer 2015;23:1081-90.

57. Elaldi R, Roussel, LM, Gal, J, et al. Correlations between long-term quality of life and patient needs and concerns following head and neck cancer treatment and the impact of psychological distress. A multicentric cross-sectional study. European Archives of Oto-Rhino-Laryngology 2020. http://dx.doi.org/10.1007/s00405-020-06326-8.
58. Elfring T, Boliek, CA, Winget, M, et al. The relationship between lingual and hypoglossal nerve function and quality of life in head and neck cancer. Journal of oral rehabilitation 2014;41:133-40.
59. Epstein JB, Emerton, S, Kolbinson, DA, et al. Quality of life and oral function following radiotherapy for head and neck cancer. Head and Neck 1999;21:1-11.
60. Epstein JB, Robertson, M, Emerton, S, et al. Quality of life and oral function in patients treated with radiation therapy for head and neck cancer. Head Neck 2001;23:389-98.
61. Erkal EY, Canoğlu, D, Kaya, A, et al. Assessment of early and late dysphagia using videofluoroscopy and quality of life questionnaires in patients with head and neck cancer treated with radiation therapy. Radiat Oncol 2014;9:137.
62. Ettl T, Weindler, J, Gosau, M, et al. Impact of radiotherapy on implant-based prosthetic rehabilitation in patients with head and neck cancer: A prospective observational study on implant survival and quality of life—Preliminary results. Journal of Cranio-Maxillofacial Surgery 2016;44:1453-62.
63. Evensen JF, Bjordal, K, Knutsen, BH, et al. Side effects and quality of life after inadvertent radiation overdosage in brachytherapy of head-and-neck cancer. International Journal of Radiation Oncology Biology Physics 2002;52:944-52.
64. Fang FM, Chien, CY, Kuo, SC, et al. Changes in quality of life of head-and-neck cancer patients following postoperative radiotherapy. Acta Oncologica 2004;43:571-8.
65. Fang FM, Tsai, WL, Chien, CY, et al. Changing quality of life in patients with advanced head and neck cancer after primary radiotherapy or chemoradiation. Oncology 2005;68:405-13.
66. Felser S, Behrens, M, Liese, J, et al. Feasibility and Effects of a Supervised Exercise Program Suitable for Independent Training at Home on Physical Function and Quality of Life in Head and Neck Cancer Patients: A Pilot Study. Integrative Cancer Therapies 2020;19.
67. Fierz J, Bürgin, W, Mericske-Stern, R. Patients with oral tumors. Part 2: Quality of life after treatment with resection prostheses. Resection prosthetics: evaluation of quality of life. Schweizer Monatsschrift für Zahnmedizin = Revue mensuelle suisse d'odonto-stomatologie = Rivista mensile svizzera di odontologia e stomatologia / SSO 2013;123:180-91.
68. Franco P, Rampino, M, Ostellino, O, et al. Management of acute skin toxicity with Hypericum perforatum and neem oil during platinum-based concurrent chemo-radiation in head and neck cancer patients. Medical Oncology 2017;34.

69. Funk CS, Warmling, CM, Baldisserotto, J. A randomized clinical trial to evaluate the impact of a dental care program in the quality of life of head and neck cancer patients. Clinical oral investigations 2014;18:1213-9.
70. Galitis E, Droukas, V, Tzakis, M, et al. Trismus and reduced quality of life in patients with oral squamous cell carcinoma, who received post-operative radiotherapy alone or combined with chemotherapy. Forum of Clinical Oncology 2017;8:29-36.
71. Gandhi AK, Roy, S, Thakar, A, et al. Symptom Burden and Quality of Life in Advanced Head and Neck Cancer Patients: AIIMS Study of 100 Patients. Indian J Palliat Care 2014;20:189-93.
72. Goetz C, Raschka, J, Wolff, KD, et al. Hospital based quality of life in oral cancer surgery. Cancers 2020;12:1-13.
73. Goiato MC, Amoroso, AP, Silva, B, et al. The Impact of Surgery and Radiotherapy on Health-Related Quality of Life of Individuals with Oral and Oropharyngeal Carcinoma and Short-Term Follow up after Treatment. Asian Pacific journal of cancer prevention : APJCP 2020;21:1227-34.
74. Guenzel T, Walliczek-Dworschak, U, Teymoortash, A, et al. Health-related quality of life in oropharyngeal cancer survivors -A population-based study. Otolaryngologia Polska 2018;72:28-33.
75. Ham JC, van Herpen, CML, Driessen, CML, et al. Health-related quality of life of patients treated with chemoradiotherapy plus or minus prophylactic antibiotics to reduce the number of pneumonias for locally advanced head and neck cancer, the PANTAP study. Oral Oncology 2019;96:105-12.
76. Hammerlid E, Bjordal, K, Ahlner-Elmqvist, M, et al. Prospective, longitudinal quality-of-life study of patients with head and neck cancer: A feasibility study including the EORTC QLC-C30. Otolaryngology - Head and Neck Surgery 1997;116:666-73.
77. Hammerlid E, Mercke, C, Sullivan, M, et al. A prospective quality of life study of patients with oral or pharyngeal carcinoma treated with external beam irradiation with or without brachytherapy. European Journal of Cancer Part B: Oral Oncology 1997;33:189-96.
78. Hammerlid E, Bjordal, K, Ahlner-Elmqvist, M, et al. A prospective study of quality of life in head and neck cancer patients. Part I: At diagnosis. Laryngoscope 2001;111:669-80.
79. Hammerlid E, Silander, E, Hörnestam, L, et al. Health-related quality of life three years after diagnosis of head and neck cancer--a longitudinal study. Head Neck 2001;23:113-25.
80. Hammerlid E, Taft, C. Health-related quality of life in long-term head and neck cancer survivors: a comparison with general population norms. Br J Cancer 2001;84:149-56.
81. Hashemipour MA, Pooyafard, A, Navabi, N, et al. Quality of life in Iranian patients with head-and-neck cancer. J Educ Health Promot 2020;9:358.

82. Hassanein KA, Musgrove, BT, Bradbury, E. Psychological outcome of patients following treatment of oral cancer and its relation with functional status and coping mechanisms. J Craniomaxillofac Surg 2005;33:404-9.
83. Hermann GM, Iovoli, AJ, Platek, AJ, et al. A single-institution, randomized, pilot study evaluating the efficacy of gabapentin and methadone for patients undergoing chemoradiation for head and neck squamous cell cancer. Cancer 2020;126:1480-91.
84. Ho KF, Farnell, DJ, Routledge, JA, et al. Comparison of patient-reported late treatment toxicity (LENT-SOMA) with quality of life (EORTC QLQ-C30 and QLQ-H&N35) assessment after head and neck radiotherapy. Radiother Oncol 2010;97:270-5.
85. Horn D, Zittel, S, Moratin, J, et al. Prospective feasibility analysis of salvage surgery in recurrent oral cancer in terms of quality of life. Oral Oncology 2020;102.
86. Huguenin PU, Taussky, D, Moe, K, et al. Quality of life in patients cured from a carcinoma of the head and neck by radiotherapy: the importance of the target volume. Int J Radiat Oncol Biol Phys 1999;45:47-52.
87. Infante-Cossio P, Torres-Carranza, E, Cayuela, A, et al. Quality of life in patients with oral and oropharyngeal cancer. International Journal of Oral and Maxillofacial Surgery 2009;38:250-5.
88. Iwase M, Takemi, T, Manabe, M, et al. Hypercalcemic complication in patients with oral squamous cell carcinoma. International Journal of Oral and Maxillofacial Surgery 2003;32:174-80.
89. Jacobsen HC, Wahnschaff, F, Trenkle, T, et al. Oral rehabilitation with dental implants and quality of life following mandibular reconstruction with free fibular flap. Clinical oral investigations 2016;20:187-92.
90. Jellema AP, Doornaert, P, Slotman, BJ, et al. Does radiation dose to the salivary glands and oral cavity predict patient-rated xerostomia and sticky saliva in head and neck cancer patients treated with curative radiotherapy? Radiotherapy and Oncology 2005;77:164-71.
91. Jellema AP, Slotman, BJ, Doornaert, P, et al. Unilateral versus bilateral irradiation in squamous cell head and neck cancer in relation to patient-rated xerostomia and sticky saliva. Radiotherapy and Oncology 2007;85:83-9.
92. Jenewein J, Zwahlen, RA, Zwahlen, D, et al. Quality of life and dyadic adjustment in oral cancer patients and their female partners. European Journal of Cancer Care 2008;17:127-35.
93. Jensen K, Bonde Jensen, A, Grau, C. The relationship between observer-based toxicity scoring and patient assessed symptom severity after treatment for head and neck cancer. A correlative cross sectional study of the DAHANCA toxicity scoring system and the EORTC quality of life questionnaires. Radiotherapy and Oncology 2006;78:298-305.
94. Jensen K, Jensen, AB, Grau, C. A cross sectional quality of life study of 116 recurrence free head and neck cancer patients. The first use of EORTC H&N35 in Danish. Acta Oncologica 2006;45:28-37.

95. Jensen K, Jensen, AB, Grau, C. Smoking has a negative impact upon health related quality of life after treatment for head and neck cancer. Oral Oncology 2007;43:187-92.
96. Kaae JK, Johnsen, L, Hansen, CR, et al. Relationship between patient and physician-rated xerostomia and dose distribution to the oral cavity and salivary glands for head and neck cancer patients after radiotherapy. Acta Oncologica 2019;58:1366-72.
97. Kermer C, Ziya-Ghazvini, F, Poeschl, PW, et al. Two stage reconstruction with revascularized grafts after resection of retromolar and oropharyngeal carcinoma. International Journal of Oral and Maxillofacial Surgery 2004;33:554-7.
98. Kim SA, Roh, JL, Lee, SA, et al. Pretreatment depression as a prognostic indicator of survival and nutritional status in patients with head and neck cancer. Cancer 2016;122:131-40.
99. Kjeldsted E, Dalton, SO, Frederiksen, K, et al. Association between human papillomavirus status and health-related quality of life in oropharyngeal and oral cavity cancer survivors. Oral Oncology 2020;109.
100. Korfage A, Schoen, PJ, Raghoebar, GM, et al. Five-year follow-up of oral functioning and quality of life in patients with oral cancer with implant-retained mandibular overdentures. Head Neck 2011;33:831-9.
101. Korsten LHA, Jansen, F, Lissenberg-Witte, BI, et al. The course of health-related quality of life from diagnosis to two years follow-up in patients with oropharyngeal cancer: does HPV status matter? Supportive Care in Cancer 2021. http://dx.doi.org/10.1007/s00520-020-05932-w.
102. Krebber AMH, Jansen, F, Cuijpers, P, et al. Screening for psychological distress in follow-up care to identify head and neck cancer patients with untreated distress. Supportive Care in Cancer 2016;24:2541-8.
103. Kumar VV, Jacob, PC, Ebenezer, S, et al. Implant supported dental rehabilitation following segmental mandibular reconstruction- quality of life outcomes of a prospective randomized trial. Journal of Cranio-Maxillofacial Surgery 2016;44:800-10.
104. Lal P, Nautiyal, V, Verma, M, et al. Objective and subjective assessment of xerostomia in patients of locally advanced head-and-neck cancers treated by intensity-modulated radiotherapy. J Cancer Res Ther 2018;14:1196-201.
105. Landström FJ, Reizenstein, J, Adamsson, GB, et al. Long-term follow-up in patients treated with curative electrochemotherapy for cancer in the oral cavity and oropharynx. Acta Oto-Laryngologica 2015;135:1070-8.
106. Lazaridou M, Konstantinos, V, Ioannis, D, et al. Nasolabial pedicled compared with island flaps for intraoral reconstruction of oncological defects: complications, recovery of sensitivity, and assessment of quality of life. British Journal of Oral and Maxillofacial Surgery 2016;54:746-50.

107. Lazarus CL, Husaini, H, Anand, SM, et al. Tongue strength as a predictor of functional outcomes and quality of life after tongue cancer surgery. Annals of Otology, Rhinology and Laryngology 2013;122:386-97.
108. Lazarus CL, Husaini, H, Hu, K, et al. Functional outcomes and quality of life after chemoradiotherapy: Baseline and 3 and 6 months post-treatment. Dysphagia 2014;29:365-75.
109. Lee HF, Liu, HE. Prospective changes of the quality of life for patients newly diagnosed with oral cancer during the acute stage. European Journal of Oncology Nursing 2010;14:310-5.
110. Lee LY, Chen, SC, Chen, WC, et al. Postradiation trismus and its impact on quality of life in patients with head and neck cancer. Oral surgery, oral medicine, oral pathology and oral radiology 2015;119:187-95.
111. Likhterov I, Ru, M, Ganz, C, et al. Objective and subjective hyposalivation after treatment for head and neck cancer: Long-term outcomes. Laryngoscope 2018;128:2732-9.
112. Lima AG, Antequera, R, Peres, MP, et al. Efficacy of low-level laser therapy and aluminum hydroxide in patients with chemotherapy and radiotherapy-induced oral mucositis. Braz Dent J 2010;21:186-92.
113. Lindblom U, Gärskog, O, Kjellén, E, et al. Radiation-induced trismus in the ARTSCAN head and neck trial. Acta Oncologica 2014;53:620-7.
114. Llewellyn CD, McGurk, M, Weinman, J. Head and neck cancer: To what extent can psychological factors explain differences between health-related quality of life and individual quality of life? British Journal of Oral and Maxillofacial Surgery 2006;44:351-7.
115. Llewellyn CD, McGurk, M, Weinman, J. Illness and treatment beliefs in head and neck cancer: Is Leventhal's common sense model a useful framework for determining changes in outcomes over time? Journal of Psychosomatic Research 2007;63:17-26.
116. Löfstrand J, Nyberg, M, Karlsson, T, et al. Quality of Life after Free Fibula Flap Reconstruction of Segmental Mandibular Defects. Journal of Reconstructive Microsurgery 2018;34:108-20.
117. López-Jornet P, Camacho-Alonso, F, López-Tortosa, J, et al. Assessing quality of life in patients with head and neck cancer in Spain by means of EORTC QLQ-C30 and QLQ-H&N35. Journal of Cranio-Maxillofacial Surgery 2012;40:614-20.
118. Louise Kent M, Brennan, MT, Noll, JL, et al. Radiation-induced trismus in head and neck cancer patients. Supportive Care in Cancer 2008;16:305-9.
119. Low C, Fullarton, M, Parkinson, E, et al. Issues of intimacy and sexual dysfunction following major head and neck cancer treatment. Oral Oncology 2009;45:898-903.
120. Maciejewski O, Smeets, R, Gerhards, F, et al. Gender specific quality of life in patients with oral squamous cell carcinomas. Head & face medicine 2010;6:21.

121. Melissant HC, Jansen, F, Schutte, LER, et al. The course of sexual interest and enjoyment in head and neck cancer patients treated with primary (chemo)radiotherapy. Oral Oncology 2018;83:120-6.
122. Milovanović J, Andrejić, D, Jotić, A, et al. The impact of socioeconomic factors on quality of life and functional impairment in patients treated for oropharyngeal carcinoma. Vojnosanitetski Pregled 2019;76:598-606.
123. Mogadas S, Busch, CJ, Pflug, C, et al. Influence of radiation dose to pharyngeal constrictor muscles on late dysphagia and quality of life in patients with locally advanced oropharyngeal carcinoma. Strahlentherapie und Onkologie 2020;196:522-9.
124. Nagy J, Braunitzer, G, Antal, M, et al. Quality of life in head and neck cancer patients after tumor therapy and subsequent rehabilitation: An exploratory study. Quality of Life Research 2014;23:135-43.
125. Ninu MB, Miccinesi, G, Bulli, F, et al. Psychological distress and health-related quality of life among head and neck cancer patients during the first year after treatment. Tumori 2016;102:96-102.
126. Noronha V, Joshi, A, Marfatia, S, et al. Health-related quality of life in patients with metastatic, relapsed, or inoperable squamous cell carcinoma of the head and neck in India. Supportive Care in Cancer 2016;24:1595-602.
127. Nourissat A, Bairati, I, Fortin, A, et al. Factors associated with weight loss during radiotherapy in patients with stage i or II head and neck cancer. Supportive Care in Cancer 2012;20:591-9.
128. Nyqvist J, Fransson, P, Laurell, G, et al. Differences in health related quality of life in the randomised ARTSCAN study; Accelerated vs. conventional radiotherapy for head and neck cancer. A five year follow up. Radiotherapy and Oncology 2016;118:335-41.
129. Oates J, Davies, S, Roydhouse, JK, et al. The effect of cancer stage and treatment modality on quality of life in oropharyngeal cancer. Laryngoscope 2014;124:151-8.
130. Öhrn KE, Sjödén, PO, Wahlin, YB, et al. Oral health and quality of life among patients with head and neck cancer or haematological malignancies. Supportive Care in Cancer 2001;9:528-38.
131. Oliveira KG, von Zeidler, SV, Podestá, JRV, et al. Influence of pain severity on the quality of life in patients with head and neck cancer before antineoplastic therapy. BMC Cancer 2014;14.
132. Oskam IM, Verdonck-De Leeuw, IM, Aaronson, NK, et al. Quality of life as predictor of survival: A prospective study on patients treated with combined surgery and radiotherapy for advanced oral and oropharyngeal cancer. Radiotherapy and Oncology 2010;97:258-62.
133. Osthus AA, Aarstad, AKH, Olofsson, J, et al. Head and neck specific Health Related Quality of Life scores predict subsequent survival in successfully treated head and neck cancer patients: A prospective cohort study. Oral Oncology 2011;47:974-9.

134. Passchier E, Stuiver, MM, van der Molen, L, et al. Feasibility and impact of a dedicated multidisciplinary rehabilitation program on health-related quality of life in advanced head and neck cancer patients. Eur Arch Otorhinolaryngol 2016;273:1577-87.
135. Pauli N, Johnson, J, Finizia, C, et al. The incidence of trismus and long-term impact on health-related quality of life in patients with head and neck cancer. Acta Oncologica 2013;52:1137-45.
136. Pauli N, Fagerberg-Mohlin, B, Andréll, P, et al. Exercise intervention for the treatment of trismus in head and neck cancer. Acta Oncologica 2014;53:502-9.
137. Pauli N, Andréll, P, Johansson, M, et al. Treating trismus: A prospective study on effect and compliance to jaw exercise therapy in head and neck cancer. Head and Neck 2015;37:1738-44.
138. Pauli N, Svensson, U, Karlsson, T, et al. Exercise intervention for the treatment of trismus in head and neck cancer - a prospective two-year follow-up study. Acta Oncol 2016;55:686-92.
139. Petrovic I, Baser, R, Blackwell, T, et al. Long-term functional and esthetic outcomes after fibula free flap reconstruction of the mandible. Head and Neck 2019;41:2123-32.
140. Pierre CS, Dassonville, O, Chamorey, E, et al. Long-term functional outcomes and quality of life after oncologic surgery and microvascular reconstruction in patients with oral or oropharyngeal cancer. Acta Oto-Laryngologica 2014;134:1086-93.
141. Pollom EL, Wang, E, Bui, TT, et al. A prospective study of electronic quality of life assessment using tablet devices during and after treatment of head and neck cancers. Oral Oncology 2015;51:1132-7.
142. Ranta P, Kinnunen, I, Jouhi, L, et al. Long-term Quality of Life After Treatment of Oropharyngeal Squamous Cell Carcinoma. Laryngoscope 2020. http://dx.doi.org/10.1002/lary.29042.
143. Rathod S, Gupta, T, Ghosh-Laskar, S, et al. Quality-of-life (QOL) outcomes in patients with head and neck squamous cell carcinoma (HNSCC) treated with intensity-modulated radiation therapy (IMRT) compared to three-dimensional conformal radiotherapy (3D-CRT): Evidence from a prospective randomized study. Oral Oncology 2013;49:634-42.
144. Ravasco P, Monteiro-Grillo, I, Marques Vidal, P, et al. Impact of nutrition on outcome: a prospective randomized controlled trial in patients with head and neck cancer undergoing radiotherapy. Head Neck 2005;27:659-68.
145. Rhemrev R, Rakhorst, HA, Zuidam, JM, et al. Long-term functional outcome and satisfaction after radial forearm free flap reconstructions of intraoral malignancy resections. J Plast Reconstr Aesthet Surg 2007;60:588-92.
146. Rinkel RN, Verdonck-de Leeuw, IM, Langendijk, JA, et al. The psychometric and clinical validity of the SWAL-QOL questionnaire in evaluating swallowing problems experienced by patients with oral and oropharyngeal cancer. Oral Oncol 2009;45:e67-71.

147. Rogers SN, Lowe, D, Brown, JS, et al. A comparison between the University of Washington Head and Neck Disease- Specific Measure and the Medical Short Form 36, EORTC QOQ-C33 and EORTC Head and Neck 35. Oral Oncology 1998;34:361-72.
148. Rogers SN, Hannah, L, Lowe, D, et al. Quality of life 5-10 years after primary surgery for oral and oro-pharyngeal cancer. Journal of Cranio-Maxillo-Facial Surgery 1999;27:187-91.
149. Rogers SN, Lowe, D, Humphris, G. Distinct patient groups in oral cancer: A prospective study of perceived health status following primary surgery. Oral Oncology 2000;36:529-38.
150. Rogers SN, Lowe, D, Brown, JS, et al. The relationship between length of stay and health-related quality of life in patients treated by primary surgery for oral and oropharyngeal cancer. International journal of oral and maxillofacial surgery 2001;30:209-15.
151. Rogers SN, Waylen, AE, Thomas, S, et al. Quality of life, cognitive, physical and emotional function at diagnosis predicts head and neck cancer survival: analysis of cases from the Head and Neck 5000 study. European Archives of Oto-Rhino-Laryngology 2020;277:1515-23.
152. Roick J, Danker, H, Dietz, A, et al. Predictors of changes in quality of life in head and neck cancer patients: a prospective study over a 6-month period. Eur Arch Otorhinolaryngol 2020;277:559-67.
153. Ruhl CM, Gleich, LL, Gluckman, JL. Survival, function, and quality of life after total glossectomy. Laryngoscope 1997;107:1316-21.
154. Ryzek DF, Mantsopoulos, K, Künzel, J, et al. Early stage oropharyngeal carcinomas: Comparing quality of life for different treatment modalities. BioMed Research International 2014;2014.
155. Sandmæl JA, Bye, A, Solheim, TS, et al. Physical rehabilitation in patients with head and neck cancer: Impact on health-related quality of life and suitability of a post-treatment program. Laryngoscope Investigative Otolaryngology 2020;5:330-8.
156. Schiefke F, Akdemir, M, Weber, A, et al. Function, postoperative morbidity, and quality of life after cervical sentinel node biopsy and after selective neck dissection. Head and Neck 2009;31:503-12.
157. Schliephake H, Schmelzeisen, R, Schönweiler, R, et al. Speech, deglutition and life quality after intraoral tumour resection: A prospective study. International Journal of Oral and Maxillofacial Surgery 1998;27:99-105.
158. Schliephake H, Jamil, MU. Prospective evaluation of quality of life after oncologic surgery for oral cancer. International Journal of Oral and Maxillofacial Surgery 2002;31:427-33.
159. Schliephake H, Jamil, MU. Impact of intraoral soft-tissue reconstruction on the development of quality of life after ablative surgery in patients with oral cancer. Plastic and Reconstructive Surgery 2002;109:421-30.
160. Scott SI, Kathrine Ø. Madsen, A, Rubek, N, et al. Long-term quality of life & functional outcomes after treatment of oropharyngeal cancer. Cancer Medicine 2020. http://dx.doi.org/10.1002/cam4.3599.

161. Shepherd KL, Fisher, SE. Prospective evaluation of quality of life in patients with oral and oropharyngeal cancer: From diagnosis to three months post-treatment. Oral Oncology 2004;40:751-7.
162. Silveira AP, Gonçalves, J, Sequeira, T, et al. Geriatric oncology: Comparing health related quality of life in head and neck cancer patients. Head and Neck Oncology 2011;3.
163. Sjamsudin E, Maulina, T, Cipta, A, et al. Assessment of oral cancer pain, anxiety, and quality of life of oral squamous cell carcinoma patients with invasive treatment procedure. Oral and maxillofacial surgery 2018;22:83-90.
164. Sodergren SC, Husson, O, Rohde, GE, et al. Does age matter? A comparison of health-related quality of life issues of adolescents and young adults with cancer. European journal of cancer care 2018;27:e12980.
165. Söderström K, Nilsson, P, Laurell, G, et al. Dysphagia – Results from multivariable predictive modelling on aspiration from a subset of the ARTSCAN trial. Radiotherapy and Oncology 2017;122:192-9.
166. Spiegel JL, Pilavakis, Y, Weiss, BG, et al. Quality of life in patients after reconstruction with the supraclavicular artery island flap (SCAIF) versus the radial free forearm flap (RFFF). European Archives of Oto-Rhino-Laryngology 2019;276:2311-8.
167. Strömgren AS, Groenvold, M, Petersen, MA, et al. Pain characteristics and treatment outcome for advanced cancer patients during the first week of specialized palliative care. Journal of Pain and Symptom Management 2004;27:104-13.
168. Subramaniam S, Kong, YC, Chinna, K, et al. Health-related quality of life and psychological distress among cancer survivors in a middle-income country. Psycho-Oncology 2018;27:2172-9.
169. Suzuki M, Deno, M, Myers, M, et al. Anxiety and depression in patients after surgery for head and neck cancer in Japan. Palliative & supportive care 2016;14:269-77.
170. Teguh DN, Levendag, PC, Noever, I, et al. Early Hyperbaric Oxygen Therapy for Reducing Radiotherapy Side Effects: Early Results of a Randomized Trial in Oropharyngeal and Nasopharyngeal Cancer. International Journal of Radiation Oncology Biology Physics 2009;75:711-6.
171. Tesic M, Cankovic, M, Jevtic, M, et al. Validation of the oral health impact profile - 14 in patients with head and neck cancer. Medicina oral, patologia oral y cirugia bucal 2020;25:e739-e44.
172. Thor M, Olsson, CE, Oh, JH, et al. Temporal patterns of patient-reported trismus and associated mouth-opening distances in radiotherapy for head and neck cancer: A prospective cohort study. Clin Otolaryngol 2018;43:22-30.
173. Tribius S, Reemts, E, Prosch, C, et al. Global quality of life during the acute toxicity phase of multimodality treatment for patients with head and neck cancer: Can we identify patients most at risk of profound quality of life decline? Oral Oncology 2012;48:898-904.

174. Tribius S, Raguse, M, Voigt, C, et al. Residual deficits in quality of life one year after intensity-modulated radiotherapy for patients with locally advanced head and neck cancer: Results of a prospective study. Strahlenther Onkol 2015;191:501-10.
175. Tribius S, Meyer, MS, Pflug, C, et al. Socioeconomic status and quality of life in patients with locally advanced head and neck cancer. Strahlentherapie und Onkologie 2018;194:737-49.
176. Tribius S, Pazdyka, H, Tennstedt, P, et al. Prognostic factors for lymphedema in patients with locally advanced head and neck cancer after combined radio(chemo)therapy- results of a longitudinal study. Oral Oncology 2020;109.
177. Tribius S, Haladyn, S, Hanken, H, et al. Parotid sparing and quality of life in long-term survivors of locally advanced head and neck cancer after intensity-modulated radiation therapy. Strahlenther Onkol 2021;197:219-30.
178. Tschiesner U, Linseisen, E, Becker, S, et al. Content validation of the International Classification of Functioning, Disability and Health core sets for head and neck cancer: A multicentre study. Journal of Otolaryngology - Head and Neck Surgery 2010;39:674-87.
179. Tschiesner U, Schuster, L, Strieth, S, et al. Functional outcome in patients with advanced head and neck cancer: Surgery and reconstruction with free flaps versus primary radiochemotherapy. European Archives of Oto-Rhino-Laryngology 2012;269:629-38.
180. van Beek FE, Jansen, F, Mak, L, et al. The course of symptoms of anxiety and depression from time of diagnosis up to 2 years follow-up in head and neck cancer patients treated with primary (chemo)radiation. Oral Oncology 2020;102.
181. Van Cann EM, Dom, M, Koole, R, et al. Health related quality of life after mandibular resection for oral and oropharyngeal squamous cell carcinoma. Oral Oncol 2005;41:687-93.
182. van den Berg MG, Rasmussen-Conrad, EL, van Nispen, L, et al. A prospective study on malnutrition and quality of life in patients with head and neck cancer. Oral Oncol 2008;44:830-7.
183. van der Meulen IC, May, AM, de Leeuw, JR, et al. Long-term effect of a nurse-led psychosocial intervention on health-related quality of life in patients with head and neck cancer: a randomised controlled trial. Br J Cancer 2014;110:593-601.
184. van der Schroeff MP, Derks, W, Hordijk, GJ, et al. The effect of age on survival and quality of life in elderly head and neck cancer patients: A long-term prospective study. European Archives of Oto-Rhino-Laryngology 2007;264:415-22.
185. van Nieuwenhuizen AJ, Buffart, LM, van Uden-Kraan, CF, et al. Patient-reported physical activity and the association with health-related quality of life in head and neck cancer survivors. Supportive Care in Cancer 2018;26:1087-95.
186. Veldhuis D, Probst, G, Marek, A, et al. Tumor site and disease stage as predictors of quality of life in head and neck cancer: a prospective study on patients treated with surgery or combined therapy with surgery and radiotherapy or radiochemotherapy. European Archives of Oto-Rhino-Laryngology 2016;273:215-24.

187. Verdonck-de Leeuw IM, de Bree, R, Keizer, AL, et al. Computerized prospective screening for high levels of emotional distress in head and neck cancer patients and referral rate to psychosocial care. Oral Oncology 2009;45:e129-e33.
188. Verdonck-de Leeuw IM, van Bleek, WJ, Leemans, CR, et al. Employment and return to work in head and neck cancer survivors. Oral Oncol 2010;46:56-60.
189. Verdonck-De Leeuw IM, Buffart, LM, Heymans, MW, et al. The course of health-related quality of life in head and neck cancer patients treated with chemoradiation: A prospective cohort study. Radiotherapy and Oncology 2014;110:422-8.
190. Villaron C, Cury, F, Eisinger, F, et al. Telehealth applied to physical activity during cancer treatment: a feasibility, acceptability, and randomized pilot study. Supportive Care in Cancer 2018;26:3413-21.
191. Wang K, Amdur, RJ, Mendenhall, WM, et al. Impact of post-chemoradiotherapy superselective/selective neck dissection on patient reported quality of life. Oral Oncology 2016;58:21-6.
192. Wang F, Huang, W, Zhang, C, et al. Functional outcome and quality of life after a maxillectomy: a comparison between an implant supported obturator and implant supported fixed prostheses in a free vascularized flap. Clin Oral Implants Res 2017;28:137-43.
193. Wan Leung S, Lee, TF, Chien, CY, et al. Health-related Quality of life in 640 head and neck cancer survivors after radiotherapy using EORTC QLQ-C30 and QLQ-H&N35 questionnaires. BMC Cancer 2011;11.
194. Watkinson JC, Owen, C, Thompson, S, et al. Conservation surgery in the management of T1 and T2 oropharyngeal squamous cell carcinoma: The Birmingham UK experience. Clinical Otolaryngology and Allied Sciences 2002;27:541-8.
195. Yan R, Chen, X, Gong, X, et al. The association of tooth loss, toothbrushing, and quality of life among cancer survivors. Cancer Medicine 2018;7:6374-84.
196. Yokota T, Hamauchi, S, Yoshida, Y, et al. A phase II study of HMB/Arg/Gln against oral mucositis induced by chemoradiotherapy for patients with head and neck cancer. Supportive Care in Cancer 2018;26:3241-8.
197. You Q, jing, X, Fan, S, et al. Comparison of functional outcomes and health-related quality of life one year after treatment in patients with oral and oropharyngeal cancer treated with three different reconstruction methods. British Journal of Oral and Maxillofacial Surgery 2020;58:759-65.
198. Zrnc TA, Tomic, J, Tomazic, PV, et al. Complex mandibular reconstruction for head and neck squamous cell carcinoma—the ongoing challenge in reconstruction and rehabilitation. Cancers 2020;12:1-17.
