# Supplementary material for: Health-Related Quality of Life in Oral Cancer Patients: Scoping Review and Critical Appraisal of Investigated Determinants
Source: Cancers (Basel). 2021 Aug 31;13(17):4398. doi: 10.3390/cancers13174398 (PMC8431462; doi:10.3390/cancers13174398)
Supplement: Supplementary file 1 [file cancers-13-04398-s001.zip › Table S3 (Data extraction, SDG variables).pdf]

Table S3. Data extraction, SDG variables considered. Legend to Table S3: Ex = excluded; H = homogeneous; IS = incomplete/inadequate stratification; na = not available; NS = not stratified; S = stratified.

Article	OC sample	Gender	Age	Marital status/family	Comorb.	Smoking	Alcohol	Educational level	Employ. stasus / annual income	1) S/SOS/H/EX/NP	2) IS/ISOS	3) NS/NSOS	4) na	Considered var (tot 1+2)	Ignored var (tot 3+4)
Borggreven 2007	38	S	NS	S	S	na	na	na	na	3	0	1	4	3	5
Khandelwal 2017	50	S	S	na	na	na	na	na	na	2	0	0	6	2	6
Airoldi 2011	50	NS	NS	NS	na	NS	NS	NS	NS	0	0	7	1	0	8
Beck 2017	45	NS	NS	na	na	na	na	na	na	0	0	2	6	0	8
Becker 2012	50	NS	na	NS	na	na	na	NS	NS	0	0	4	4	0	8
Bozec 2009	21	S	S	na	S	na	na	na	na	3	0	0	5	3	5
Bozec 2020	48	S	S	S	Ex	S	S	S	na	7	0	0	1	7	1
Canis 2016	40	NS	NS	na	na	NS	NS	na	na	0	0	4	4	0	8
Crombie 2014	16	NS	NS	na	na	na	na	na	na	0	0	2	6	0	8
Davudov 2019	120	NS	NS	na	na	NS	na	na	na	0	0	3	5	0	8
Infante-Cossio 2009	70	NS	NS	na	na	na	na	na	na	0	0	2	6	0	8
Nordgren 2008	122	NS	NS	na	NS	na	na	na	na	0	0	3	5	0	8
Schoen 2008	41	NS	NS	na	na	na	na	na	na	0	0	2	6	0	8
Klug 2002	67	NS	H	na	na	na	na	na	na	1	0	1	6	1	7
Ferri 2020	55	NS	NS	na	na	na	na	na	na	0	0	2	6	0	8
Girod 2009	34	NS	NS	na	na	NS	na	na	na	0	0	3	5	0	8
Kovacs 2015	110	S	NS	na	na	na	na	na	na	1	0	1	6	1	7
Peisker 2016	100	NS	NS	na	na	na	na	na	na	0	0	2	6	0	8
Moubayed 2014	13	na	NS	na	na	na	na	na	na	0	0	1	7	0	8
Oskam 2013	38	S	IS	S	NS	NS	NS	na	na	2	1	3	2	3	5
Pierre 2014	37	S	S	na	S	na	na	na	na	3	0	0	5	3	5
Van Gemert 2015	37	S	IS	na	na	na	na	na	na	1	1	0	6	2	6
Oates 2008	47	na	na	na	na	na	na	na	na	0	0	0	8	0	8
Huang 2010	129	S	S	S	S	na	na	S	S	6	0	0	2	6	2
Lin 2020	22	NS	NS	na	na	NS	na	na	na	0	0	3	5	0	8
Mair 2017	225	NS	NS	na	na	na	na	na	na	0	0	2	6	0	8
Dzioba 2017	117	NS	NS	na	na	na	na	na	na	0	0	2	6	0	8
Kessler 20024	41	NS	NS	na	na	na	na	na	na	0	0	2	6	0	8
Yoshimura 2009	20	S	S	na	na	na	na	na	na	2	0	0	6	2	6
Petruson 2005	30	NS	NS	na	na	na	na	na	na	0	0	2	6	0	8
