# Supplementary material for: Health-Related Quality of Life in Oral Cancer Patients: Scoping Review and Critical Appraisal of Investigated Determinants
Source: Cancers (Basel). 2021 Aug 31;13(17):4398. doi: 10.3390/cancers13174398 (PMC8431462; doi:10.3390/cancers13174398)
Supplement: Supplementary file 1 [file cancers-13-04398-s001.zip › Table S4 (Data extraction, DT variables).pdf]

Table S4. Data extraction, DT variables considered.Legend to Table S4: CT = adjuvant/definitive chemotherapy; Ex = excluded; H = homogeneous; IS = incomplete/inadequate stratification; ISOS = incomplete/inadequate stratification by oral subsites; na = not available; nCT = neoadjuvant chemotherapy; ND = neck dissection; NP = not present; nRT = neoadjuvant radiotherapy; NS = not stratified; NSOS = not stratified by oral subsites; RT = adjuvant/definitive radiotherapy; S = stratified; SOS = stratified by oral subsites.

Article	OC sample	Site	Tumor stage	Mand. Resec.	Extent of surg. resec.	Surg. approach	ND	Reconstruction	nRT*	RT	nCT	CT	Synchronous lesions	Recurrency/ metachronous lesions	Major postsurg. complications	Secondary surg. needed	1) S/SOS/ H/EX/ NP	2) IS/ISOS	3) NS/NSOS	4) na	Considered var (tot 1+2)	Ignored var (tot 3+4)
Borggreven 2007	38	NSOS	IS	NS	na	NS	NS	H	NS	NS	na	na	NS	NS	na	na	1	1	8	5	2	13
Khandelwal 2017	50	ISOS	S	na	na	na	na	NS	na	NS	na	NS	na	na	na	na	1	1	3	10	2	13
Airoldi 2011	50	NSOS	NS	na	na	na	NS	H	na	H	na	na	na	na	na	na	2	0	3	10	2	13
Beck 2017	45	NSOS	IS	IS	na	na	na	IS	na	na	na	na	na	Ex	na	Ex	2	3	1	9	5	10
Becker 2012	50	ISOS	IS	S	na	na	IS	IS	NS	NS	na	na	na	NS	na	NS	1	4	4	6	5	10
Bozec 2009	21	NSOS	IS	na	na	NS	na	H	na	S	na	NS	na	Ex	IS	na	3	2	3	7	5	10
Bozec 2020	48	NSOS	IS	na	na	na	na	NS	na	S	na	na	na	na	na	na	1	1	2	11	2	13
Canis 2016	40	H	H	NP	H	H	IS	S	na	H	na	H	Ex	na	NP	NS	10	1	1	3	11	4
Crombie 2014	16	NSOS	NS	NS	na	na	IS	NS	na	NS	na	NS	na	NS	NS	NS	0	1	9	5	1	14
Davudov 2019	120	NSOS	na	H	na	na	na	S	NS	na	NS	na	na	na	na	na	2	0	3	10	2	13
Infante-Cossio 2009	70	NSOS	IS	na	na	na	na	na	NP	S	NP	S	na	Ex	na	na	5	1	1	8	6	9

Nordgren 2008	122	NSOS	NS	na	na	na	na	na	na	S	na	na	na	na	na	na	1	0	2	12	1	14
Schoen 2008	41	NSOS	NS	na	na	na	na	na	na	S	na	na	na	NS	na	na	1	0	3	11	1	14
Klug 2002	67	ISOS	S	S	na	na	IS	NS	H	na	H	na	na	Ex	na	na	5	2	1	7	7	8
Ferri 2020	55	H	H	NP	H	S	na	IS	NP	NS	NP	NP	NP	NP	NP	NP	12	1	1	1	13	2
Girod 2009	34	NSOS	na	na	na	na	na	S	IS	IS	na	na	na	na	S	na	2	2	1	10	4	11
Kovacs 2015	110	SOS	NS	na	na	na	S	IS	NS	S	NS	S	na	na	na	na	4	1	3	7	5	10
Peisker 2016	100	NSOS	na	na	na	na	na	NS	na	NS	na	na	na	na	na	na	0	0	3	12	0	15
Moubayed 2014	13	NSOS	na	H	NS	na	na	S	na	NS	na	na	na	na	na	na	2	0	3	10	2	13
Oskam 2013	38	NSOS	IS	na	na	na	na	NS	na	NS	na	na	na	na	na	na	0	1	3	11	1	14
Pierre 2014	37	SOS	S	S	NS	na	na	IS	NS	NS	na	na	na	Ex	IS	na	4	2	3	6	6	9
Van Gemert 2015	37	SOS	NS	H	IS	na	IS	IS	na	S	na	na	na	Ex	Ex	na	5	3	1	6	8	7
Oates 2008	47	NSOS	NS	na	na	na	na	na	na	na	na	na	na	Ex	na	na	1	0	2	12	1	14
Huang 2010	129	NSOS	IS	na	na	na	na	na	IS	IS	IS	IS	na	NP	na	na	1	5	1	8	6	9
Lin 2020	22	H	NS	NP	NS	H	na	S	na	NS	na	na	na	na	NP	na	5	0	3	7	5	10
Mair 2017	225	H	H	NP	na	H	IS	H	NP	S	NP	S	na	S	na	na	10	1	0	4	11	4
Dzioba 2017	117	ISOS	NS	NS	NS	NS	na	NS	na	S	na	S	na	NS	na	na	2	1	6	6	3	12
Kessler 2004	41	ISOS	NS	NS	na	na	NS	IS	S	S	S	NP	na	Ex	NS	NS	5	2	5	3	7	8
Yoshimura 2009 ***	20	ISOS	IS	NP	NP	NP	NP	NP	NP	NP	NP	NP	NP	Ex	NP	NP	13	2	0	0	15	0
Petruson 2005 ***	30	H	NS	NP	NP	NP	NP	NP	NP	NP	NP	NP	na	na	NP	na	11	0	1	3	11	4

* "incomplete/inadequate stratification" means that no results were stratified without distinction between neoadjuvant or adjuvant RT.** "incomplete/inadequate stratification" means that just some of major postsurgical complications were excluded, “Not present” means that the authors stated that complications did not occur in

the screened population, while "Excluded" means that complications occurred in the screened population and they were excluded from the sample considered for data analysis. *** Study on patients treated by non-surgical therapies.
